# Network Pharmacology-Based Study on the Mechanism of Aloe Vera for Treating Cancer

**DOI:** 10.1155/2021/6077698

**Published:** 2021-12-01

**Authors:** Jing Xie, Jun Wu, Sihui Yang, Huaijun Zhou

**Affiliations:** ^1^Nanjing Drum Tower Hospital Clinical College of Traditional Chinese and Western Medicine, Nanjing University of Chinese Medicine, Nanjing 210000, China; ^2^Nanjing Drum Tower Hospital, The Affiliated Hospital of Nanjing University Medical School, Nanjing 210000, China

## Abstract

**Background:**

Aloe vera has long been considered an anticancer herb in different parts of the world.

**Objective:**

To explore the potential mechanism of aloe vera in the treatment of cancer using network pharmacology and molecule docking approaches.

**Methods:**

The active ingredients and corresponding protein targets of aloe vera were identified from the TCMSP database. Targets related to cancer were obtained from GeneCards and OMIM databases. The anticancer targets of aloe vera were obtained by intersecting the drug targets with the disease targets, and the process was presented in the form of a Venn plot. These targets were uploaded to the String database for protein-protein interaction (PPI) analysis, and the result was visualized by Cytoscape software. Go and KEGG enrichment were used to analyze the biological process of the target proteins. Molecular docking was used to verify the relationship between the active ingredients of aloe vera and predicted targets.

**Results:**

By screening and analyzing, 8 active ingredients and 174 anticancer targets of aloe vera were obtained. The active ingredient-anticancer target network constructed by Cytoscape software indicated that quercetin, arachidonic acid, aloe-emodin, and beta-carotene, which have more than 4 gene targets, may play crucial roles. In the PPI network, AKT1, TP53, and VEGFA have the top 3 highest values. The anticancer targets of aloe vera were mainly involved in pathways in cancer, prostate cancer, bladder cancer, pancreatic cancer, and non-small-cell lung cancer and the TNF signaling pathway. The results of molecular docking suggested that the binding ability between TP53 and quercetin was the strongest.

**Conclusion:**

This study revealed the active ingredients of aloe vera and the potential mechanism underlying its anticancer effect based on network pharmacology and provided ideas for further research.

## 1. Introduction

*Aloe vera* (L.) Burm.f (synonym *A. barbadensis* Mill.), a traditional herb belonging to the Liliaceae family, has been used around the world for a long time [1]. It contains various active compounds, including anthraquinones, naphthalenones, and polysaccharides [2]. Aloe vera was praised by Egyptians as the plant of immortality and by Greek physicians as a universal panacea for all diseases and has been mixed into various concoctions by some people to treat malignant tumors, even those considered advanced [3]. Meanwhile, the safety of aloe vera extract is controversial. Although studies have clearly confirmed aloe vera extract containing high proportions of aloin has the risk of causing intestinal tumors [4, 5], the significant anticancer effects of aloin at low concentrations and other active ingredients in aloe vera are difficult to ignore [6–8]. In recent years, increasing studies have been conducted on the potential therapeutic activities of aloe vera, especially on antineoplastic activity [9]. It has been reported that aloe vera has inhibitory effects on many cancers, including colorectal cancer, breast cancer, and cervical cancer [10–12]. However, the therapeutic functions of aloe vera against cancer have not been evaluated systematically. Therefore, the researchers hope to provide a theoretical basis for the research and development of new cancer drugs by exploring the anticancer ingredients of aloe vera and the related mechanisms.

Network pharmacology is a newly developed interdisciplinary subject of drug system research based on artificial intelligence and big data [13]. It is widely used in the discovery of drug active compounds and the interpretation of the overall mechanism of action, providing new scientific and technological support for clinical drug use and new drug research and development [14]. Nowadays, an increasing number of researchers are using network pharmacology to elucidate the mechanism of action of natural drugs with complex components in treating diseases [15–17].

**Figure 1:**
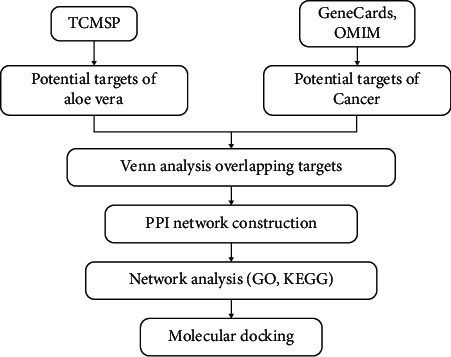
Flowchart of the network pharmacology analysis process.

In this report, network pharmacology was performed to reveal the active ingredients, therapeutic targets, and pharmacological mechanisms of aloe vera for treating cancer. The predictions of the relationship between active ingredients and key targets were verified by molecular docking. The flowchart of this investigation is presented in Figure 1.

## 2. Materials and Methods

### 2.1. Establishing the Database of Aloe Vera and Cancer

The TCMSP (http://tcmspw.com/) is a systems pharmacology platform that consists of information about Chinese herbs, including ingredients, targets, associated diseases, and ADME properties [18]. “Aloe” was used as the keyword to get the ingredients of aloe vera. The active ingredients should meet pharmacokinetic ADME criteria [19]. The related protein targets of aloe vera were obtained by searching and merging the related targets of each effective compound in TCMSP. The gene names of targets were standardized in the UniProt database (https://www.uniprot.org), and only the genes that have been “Reviewed” and belong to “Human” were left [20]. “Cancer” was used as the keyword to extract the cancer-related genes from the GeneCards (http://www.GeneCards.org) and OMIM (https://omim.org/) databases.

### 2.2. Obtaining the Anticancer Targets of Aloe Vera

Venny online tool (https://bioinfogp.cnb.csic.es/tools/venny/) was used to draw a Venn diagram to compare the targets of aloe vera and cancer. The overlap of the diagram represents the anticancer targets of aloe vera. The network of active ingredients of aloe vera and the corresponding targets was constructed and analyzed by using the software Cytoscape3.6.0. The nodes in the network represented molecules or genes, which were distinguished by different shapes, and the line between one molecule and one gene meant they were related.

### 2.3. Construction and Analyses of the PPI Network

The PPI network was obtained by uploading the anticancer targets of aloe vera to the STRING platform (https://string-db.org) with “*Homo sapiens*” chosen in the Organism box [21]. After selecting the “Medium Confidence (0.400)” in the Basic Setting column and “Hide disconnected nodes in the network” in the Advanced Setting column, the TSV format file was exported. The visual analysis of the PPI network was constructed after importing the TSV format file into Cystoscape software [22].

### 2.4. GO and KEGG Enrichment Analysis

GO and KEGG pathway enrichment analysis was used to reveal the biological function of the anticancer targets of aloe vera. The GO analysis includes molecular function, biological process, and cellular component. The anticancer targets of aloe vera were uploaded into the DAVID platform (https://david.ncifcrf.gov/) to obtain the database of GO functions and KEGG pathway. Sorted in descending order of *p* value, the top 10 data of GO analysis were visualized via GraphPad Prism 7 and the top 10 pathways were presented in the form of an enrichment dot bubble by uploading the data to the Bioinformatics platform (http://www.bioinformatics.com.cn/).

### 2.5. Verification by Molecular Docking Simulation

The network pharmacology revealed potential active ingredients of aloe vera and the key targets and pathways of aloe vera against cancer. The active compounds of aloe vera which have more than 4 gene targets were selected as the ligands and their 3D structures were searched in the PubChem database (https://pubchem.ncbi.nlm.nih.gov) and prepared using the Chem3D software. Then, the ligands were saved in the format of PDBQT after adding hydrogens, computing Gasteiger charges, detecting the root, and choosing rotatable bonds by using AutoDock Tools. Targets with the top three highest values in the PPI network were selected to be the protein receptors, and the 3D structures were extracted from the Protein Data Bank (PDB) database (http://www.rcsb.org/) [23]. The Grid Box parameters obtained by GetBox Plugin in PyMOL software were as follows: AKT (PDB ID: 1UNQ), target center *x* = 15.2, center *y* = 24.4, center *z* = 16.3, size *x* = 15.5, size *y* = 18.7, size *z* = 19.8, and spacing = 0.375 Å; TP53 (PDB ID: 3ZME), target center *x* = 91.9, center *y* = 94.1, center *z* = -45.7, size *x* = 14.0, size *y* = 20.4, size *z* = 17.2, and spacing = 0.375 Å; and VEGFA (PDB ID: 6ZFL), target center *x* = 22.9, center *y* = 19.4, center *z* = 2.2, size *x* = 13.7, size *y* = 15.6, size *z* = 15.7, and spacing = 0.375 Å. After removing water in the PyMOL software and adding hydrogens, computing charges, and adding AssignAD4 type atoms using AutoDock Tools, the receptors were saved in the PDBQT format. Molecular docking was performed via Vina, and during the docking process, all residues were kept rigid. The results were shown in the form of Vina score (affinity (kcal/mol)) [24], which represents the binding ability between the receptor and the ligand. The lower the affinity, the higher the stability of the ligand-receptor binding [25].

## 3. Results

### 3.1. Establishing the Database of Aloe Vera and Cancer

By using “Aloe” as the keyword in the TCMSP, 53 ingredients were retrieved. By setting the filter criteria that OB ≥ 30 and DL ≥ 0.18, 8 active ingredients were achieved (Table 1). The 239 related targets corresponding to the 8 active ingredients were retrieved from the TCMSP, and 181 genes were acquired after standardizing the name in the UniProt database and deleting the duplicates. In the GeneCards and OMIM disease database, “cancer” was used as the keyword. The genes with the relevance score greater than 1 in GeneCards were selected. After deleting the duplicates, 10594 cancer-related genes were obtained.

### 3.2. Acquisition of the Venn Diagram and Construction of the Component-Target Network

**Figure 2:**
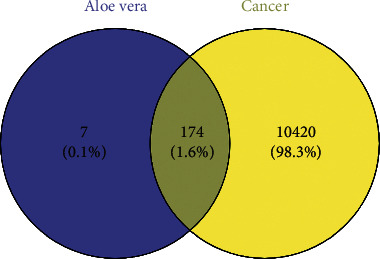
Venn diagram of targets of aloe vera and cancer. Blue circle: targets of aloe vera; yellow circle: targets of cancer.

**Figure 3:**
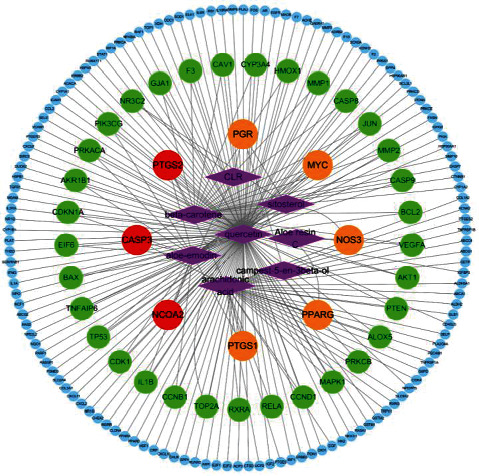
Active ingredient-anticancer target network of aloe vera. Diamond nodes: active ingredients of aloe vera; circular nodes: targets of the active ingredients.

A Venn diagram was acquired by uploading the targets of aloe vera and cancer, respectively, to the Venny platform (Figure 2). The result clearly showed that 174 of the 181 genes associated with aloe vera were also associated with cancer. The active ingredient-anticancer target network was constructed via Cytoscape, network analysis software (Figure 3). The network has 182 nodes and 226 edges. The purple diamond nodes in the middle of the network represented 8 active ingredients of aloe vera, and the 174 circular nodes surrounding the active ingredients represented the anticancer targets corresponding to the ingredients. Three target nodes associated with 4 active compounds were marked with red, 5 target nodes linked to 3 corresponding compounds were marked with orange, the target nodes related to 2 compounds were marked with green, and the target nodes only associated with 1 active compound were marked with blue. As can be seen from the figure, most of the active ingredients of aloe vera had multiple targets, and many targets corresponded to multiple active ingredients of aloe vera, which reflected that aloe vera is a multicomponent multitarget drug.

### 3.3. Construction and Analyses of the PPI Network

**Figure 4:**
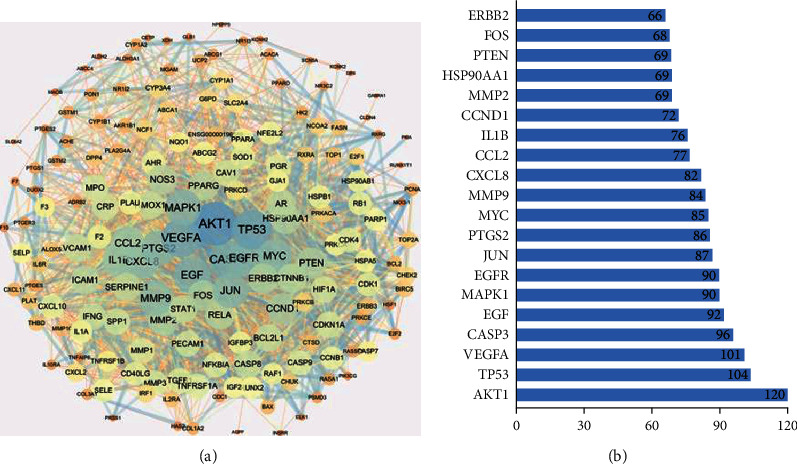
(a) PPI network of potential targets. (b) Bar chart of the top 20 genes according to degree. The *X*-axis is the degree value, and the *Y*-axis is the gene name.

The PPI network of potential targets was constructed by entering 179 anticancer targets into the STRING database and visualized by Cytoscape software. The PPI network involved 172 nodes and 2781 edges (Figure 4(a)). With the increase of node degree, the size of map nodes becomes larger and the color of map nodes changes from orange to blue. As the combined score increased, the map edge size became thicker and the map edge color turned from orange to blue. The top 20 genes according to degree were shown in a bar chart (Figure 4(b)). The degree value corresponding to each gene was plotted on each bar. Complete target analysis including degree value is shown in Table S1.

### 3.4. GO Enrichment Analysis

**Figure 5:**
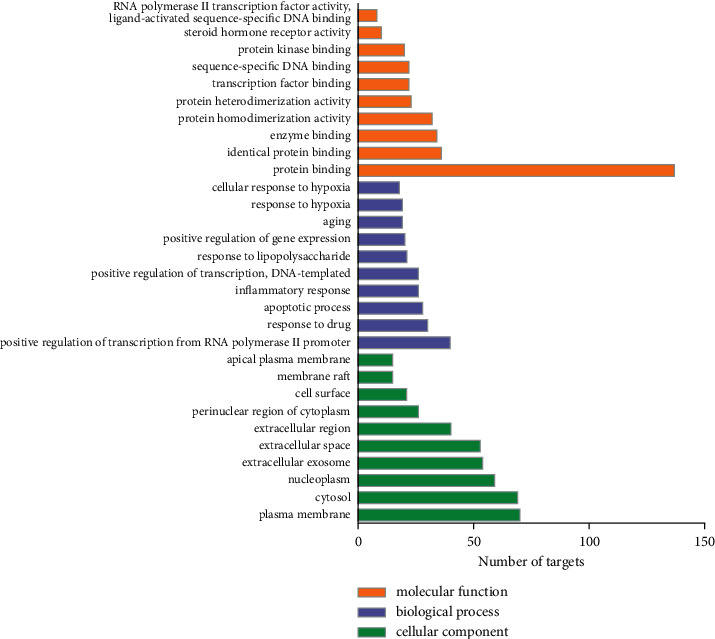
GO analysis of anticancer targets of aloe vera. The *X*-axis is the number of the targets involved in the GO analysis, and the *Y*-axis is the molecular function, biological process, or cellular component.

To further analyze the cancer targets of aloe vera, GO enrichment was performed. The top 10 representative enrichment terms, sorted by count in descending order, are displayed in Figure 5. Orange bars represented molecule function. Of all 174 potential genes, 137 genes were involved in protein binding. Bars with the color of blue represented the biological process, which mainly consisted of positive regulation of transcription from RNA polymerase II promoter (40/174) and response to drug (30/174). The cellular component shown in green bars mainly included the plasma membrane (70/174), cytosol (69/174), nucleoplasm (59/174), and extracellular exosome (54/174).

### 3.5. KEGG Pathway Enrichment Analysis

**Figure 6:**
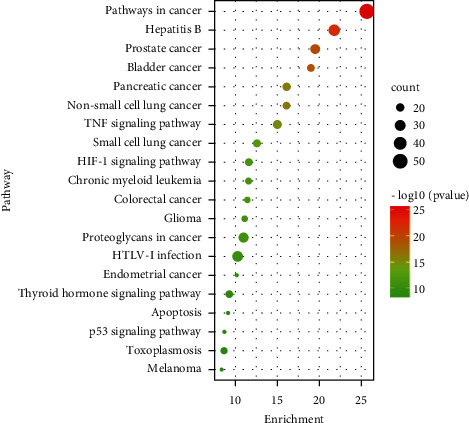
KEGG enrichment analysis (Top 20). The *X*-axis is the enrichment gene count, and the *Y*-axis is the KEGG pathway. Bubble size represents the number of genes involved in the KEGG enrichment. Color represents –log 10 (*p* value).

By uploading 174 anticancer targets to the DAVID platform, 75 pathways with *p* value <0.001 were obtained. The top 10 pathways were shown by an enrichment dot bubble in Figure 6. The pathway enrichment result indicated that the anticancer targets of aloe vera may involve in many cancers, including prostate cancer, bladder cancer, pancreatic cancer, etc. TNF, HIF-1, and p53 signaling pathways and apoptosis may play important roles in the mechanisms of aloe vera against cancer.

### 3.6. Molecular Docking

**Figure 7:**
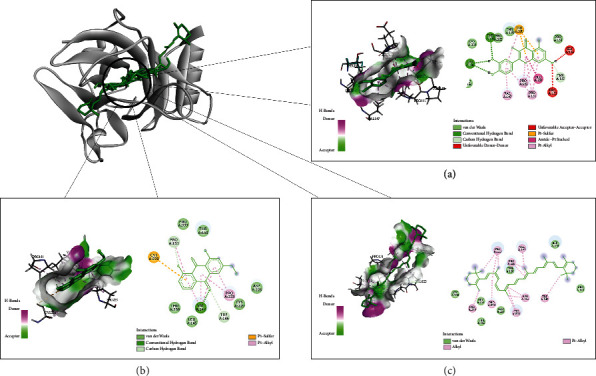
Molecule docking results for interaction of TP53 with quercetin (a), aloe-emodin (b), and beta-carotene (c). Ligand docked in the binding pocket of TP53 is placed at the left of each solid box, and 2D ligand interaction diagram is shown at the right. The ligands are shown in green.

AKT1, TP53, and VEGFA with the top 3 largest values in the PPI network were used as the receptors, and quercetin, arachidonic acid, aloe-emodin and beta-carotene, which have more than 4 gene targets, were chosen as the ligands. The ligands and receptors were prepared by using the PubChem database, PDB database, Chem3D software, PyMOL software, and AutoDock Tools. To verify the prediction, the four compounds were docked to three targets via AutoDock Vina. The result was presented in the form of affinity value (Table 2). The complete output of molecular docking is shown in Table S2. Among the three ligands, TP53 was the easiest to bind to the ligands, while VEGFA had the worst binding ability. The affinity of quercetin binding to TP53 was the lowest with the value of −8.2 kcal/mol. The binding ability between VEGFA and beta-carotene was the weakest with the value of −1.1 kcal/mol. The three target-compound pairs with the highest affinity, namely, TP53-quercetin, TP53-aloe-emodin, and TP53-beta-carotene, were selected for visualization, as shown in Figure 7.

## 4. Discussion

Aloe vera, as one of the oldest medical plants, has long been used to reduce inflammation, treat constipation, treat skin diseases, and speed wound healing [1]. At the same time, some people still believe that aloe vera has anticancer effects and use it in clinics. In the last decades, anticancer drugs have been developed endlessly and nearly half of them were unregulated natural products or their semisynthetic derivatives [26]. The lower side effects of natural phytochemicals seem to be their main advantage as anticancer agents. As a tropical xerophytic plant, aloe vera can cope with the common problems such as lack of rain, low groundwater table, and soil degradation in the tropics. It not only has a high benefit cost ratio but also can provide stable income for farmers [27]. Hence, it is of great significance to investigate the anticancer effect of aloe vera in medicine, economics, and sociology. Although increasing studies have been conducted on the antitumor effects of aloe vera and its active ingredients, the underlying mechanisms have not been systematically revealed. In this study, network pharmacology, which is an effective and economical approach for systematic therapeutic research, was performed to analyze the mechanism of aloe vera in the treatment of cancer.

In the TCMSP database, 8 active ingredients of aloe vera were screened out and then the 181 related targets were obtained by collecting and screening. The Venn diagram showed that 174 of the 181 targets associated with aloe vera were included in the cancer targets, suggesting that most of the relevant targets in aloe vera were involved in antitumor effects. The PPI network showed the interaction between various targets of aloe vera. Among these targets, AKT1 (120), TP53 (104), and VEGFA (101) with the top 3 highest degree values may be the main anticancer targets. Molecular docking was performed to further validate the possible interaction between the key targets and active molecules. AKT1 is a known oncogene which plays a direct role in cancer [28]. The protein encoded by AKT1 is a member of the serine-threonine protein kinase family, and it affects many biological processes, such as metabolism, proliferation, and angiogenesis [29]. The result of molecular docking indicated that quercetin, aloe-emodin, and beta-carotene may play an anticancer role by directly binding to AKT1. TP53, as a gene prone to frequent mutations, plays an anticancer role in a variety of tumor types. TP53 is closely related to the cell cycle, and the main biological outcome observed after TP53 activation is cell cycle arrest, which prevents the accumulation of damaged DNA and reduces genomic instability [30]. According to the molecular docking result, quercetin binding to TP53 exhibited the highest binding energy. Arachidonic acid, aloe-emodin, and beta-carotene also have good binding ability to TP53. TP53 may be the most important anticancer target of aloe vera. VEGFA belongs to the PDGF/VEGF growth factor family, which is upregulated in many known tumors and involved in processes including proliferation, migration, apoptosis, and angiogenesis [31]. VEGFA had the worst binding activity to beta-carotene and cannot bind well with quercetin, arachidonic acid, and aloe-emodin, which suggested that VEGFA may not be the direct target of aloe vera in cancer treatment.

The results of KEGG pathway analysis revealed the anticancer pathways of aloe vera. Ten of the top 20 pathways were directly related to cancer, including prostate cancer, bladder cancer, pancreatic cancer, non-small-cell lung cancer, small-cell lung cancer, chronic myeloid leukemia, colorectal cancer, glioma, endometrial cancer, and melanoma, which were involved in many systems, such as the urinary system, digestive system, respiratory system, and blood system. The results indicated that the anticancer targets of aloe vera play important roles in treating many cancers and aloe vera may have great potential in the treatment of cancer. Some of the potential targets were also involved in the TNF, HIF-1, and p53 signaling pathway and apoptosis. The TNF signaling pathway plays a critical role in various processes, including cell proliferation, differentiation, apoptosis, regulating immune response, and inducing inflammation [32]. There are 23 anticancer targets of aloe-emodin included in the TNF signaling pathway, including TNF-R1 and TNF-R2, which mediate most of the TNF-induced cellular responses [33]. Aloe vera has 19 anticancer targets involved in the HIF-1 signaling pathway, which is closely associated with hypoxia and involved in several key aspects of cancer biology, including immortalization, cellular dedifferentiation, and genetic instability [34]. The pathway of apoptosis contains 14 anticancer targets of aloe vera, including initiators of apoptosis (CASP8 and CASP 9) and effectors of apoptosis (CASP3 and CASP7) [35]. There were 14 anticancer targets of aloe vera included in the P53 signaling pathway, which is closely related to cell apoptosis, cell senescence, and cell cycle arrest [36]. Therefore, aloe vera has important value in the treatment of many cancers, such as prostate cancer, bladder cancer, and pancreatic cancer, and plays the anticancer role through one or more mechanisms.

## 5. Conclusions

In this study, 8 active ingredients and 179 potential targets of aloe vera were extracted for constructing the PPI network and performing GO and KEGG analysis. The binding potential between the active compounds and potential targets was evaluated by molecular docking. This study systematically elucidated the relationship between aloe vera and cancer at the molecular level, which indicated that aloe vera has great potential as an anticancer agent for drug development. Moreover, further in vitro and in vivo experiments should be performed to confirm the results obtained in this study.

## Figures and Tables

**Table 1:** Potential active ingredients of aloe vera.

Molecule ID	Molecule name	OB (%)	DL
MOL001439	Arachidonic acid	45.57	0.2
MOL002773	beta-Carotene	37.18	0.58
MOL000359	Sitosterol	36.91	0.75
MOL000953	CLR	37.87	0.68
MOL000471	Aloe-emodin	83.38	0.24
MOL005043	Campest-5-en-3beta-ol	37.58	0.71
MOL005051	Aloe resin C	34.99	0.5
MOL000098	Quercetin	46.43	0.28

**Table 2:** Vina score of molecular docking.

Compound	Affinity (kcal/mol)		
	AKT1	TP53	VEGFA
Quercetin	−6.0	−8.2	−5.6
Arachidonic acid	−4.8	−6.2	−3.9
Aloe-emodin	−6.1	−8.0	−5.9
beta-Carotene	−7.0	−7.4	−1.1

## Data Availability

The data used to support the findings of this study are included within the article.

## Abbreviations

TCMSP: Traditional Chinese Medicine Database and Analysis Platform
ADME: Absorption, distribution, metabolism, and excretion
GO: Gene Ontology
KEGG: Kyoto Encyclopedia of Genes and Genomes
DAVID: Database for Annotation, Visualization, and Integrated Discovery
OB: Oral bioavailability
DL: Drug-likeliness
TNF: Tumor necrosis factor
HIF-1: Hypoxia-inducible factor-1
AKT1: AKT serine/threonine kinase 1
TP53: Tumor protein P53
VEGFA: Vascular endothelial growth factor A.

## Supplementary Materials

Supplementary Materials Supplementary Table S1: target analysis including degree values. Supplementary Table S2: complete output of molecular docking.
