# Supplementary material for: Network Pharmacology-Based Study on the Mechanism of Aloe Vera for Treating Cancer
Source: Evid Based Complement Alternat Med. 2021 Dec 1;2021:6077698. doi: 10.1155/2021/6077698 (PMC8654547; doi:10.1155/2021/6077698)
Supplement: Supplementary Materials — Supplementary Table S1: target analysis including degree values. Supplementary Table S2: complete output of molecular docking. [file 6077698.f1.zip › 6077698.f1/Table S1.pdf]

Betweenness Centrality	ClosenessCentrality	ClusteringCoefficient	Degree	name	Neighborhood Connectivity
0.075704	0.75431	0.29944	120	AKT1	41.4
0.039402	0.7	0.354182	104	TP53	44.875
0.029795	0.697211	0.385149	101	VEGFA	46.66337
0.030421	0.683594	0.395395	96	CASP3	47.23958
0.048114	0.673077	0.382465	92	EGF	46.54348
0.034214	0.665399	0.396255	90	MAPK1	47.43333
0.031256	0.670498	0.394757	90	EGFR	47.04444
0.017522	0.660377	0.443197	87	JUN	50.02299
0.024133	0.652985	0.421614	86	PTGS2	48.60465
0.017293	0.650558	0.42465	85	MYC	48.37647
0.016233	0.648148	0.458979	84	MMP9	50.16667
0.015513	0.645756	0.456188	82	CXCL8	50.08537
0.021994	0.629496	0.442242	77	CCL2	48.11688
0.013468	0.62724	0.450175	76	IL1B	48.52632
0.009694	0.625	0.498435	72	CCND1	51.31944
0.0113	0.614035	0.537937	69	MMP2	54.30435
0.03194	0.618375	0.410912	69	HSP90AA1	47.2029
0.01346	0.614035	0.464621	69	PTEN	49.47826
0.010058	0.614035	0.513608	68	FOS	53.70588
0.024205	0.614035	0.487646	66	ERBB2	50.62121
0.01434	0.609756	0.440385	65	NOS3	49.56923
0.010335	0.601375	0.496672	63	PPARG	53.61905
0.00857	0.599315	0.513485	62	CTNNB1	52.91935
0.006896	0.599315	0.528292	62	RELA	55.01613
0.009394	0.601375	0.482514	61	AR	50.86885
0.004908	0.59727	0.585246	61	ICAM1	55
0.007236	0.589226	0.563998	59	SERPINE1	54.27119
0.005971	0.587248	0.53781	58	HMOX1	54.98276
0.009443	0.585284	0.506266	57	STAT1	53.82456
0.004459	0.583333	0.587093	57	VCAM1	54.07018
0.00521	0.583333	0.578571	56	CASP8	56.17857
0.003269	0.589226	0.620875	55	BCL2L1	58.21818
0.00293	0.57947	0.65269	54	IFNG	58.5
0.006917	0.571895	0.528658	52	CRP	51.25
0.00949	0.57377	0.528658	52	MPO	51.26923
0.003951	0.57947	0.602353	51	HIF1A	59.72549
0.003267	0.571895	0.623529	51	CDKN1A	57.07843
0.00409	0.575658	0.614118	51	SPP1	58.58824
0.012804	0.57947	0.505098	51	CAV1	54.92157
0.003728	0.568182	0.651596	48	MMP1	58.95833
0.002443	0.570033	0.672872	48	PECAM1	60.89583
0.003871	0.559105	0.634598	47	MMP3	57.44681
0.00154	0.564516	0.72988	47	TGFB1	63.10638
0.002605	0.566343	0.649399	47	TNFRSF1A	59.34043
0.003684	0.571895	0.593237	46	PGR	57.43478

0.001399	0.560897	0.69596	45	CASP9	61.37778
0.003252	0.55205	0.571717	45	NFKBIA	55.88889
0.004926	0.559105	0.574747	45	CDK4	51.42222
0.007385	0.55205	0.445183	43	SOD1	49.88372
0.00176	0.55205	0.702671	42	CXCL10	57.47619
0.00517	0.553797	0.520325	42	AHR	54.78571
0.003554	0.553797	0.57561	41	CDK1	50.92683
0.002739	0.550314	0.628205	40	PLAU	59.1
0.002805	0.546875	0.657692	40	CD40LG	57.35
0.005603	0.55205	0.471795	40	F2	50.325
0.00134	0.550314	0.753036	39	CCNB1	60.69231
0.001851	0.545171	0.641536	38	RB1	56.21053
7.03E-04	0.538462	0.771772	37	SELE	59.43243
0.003485	0.546875	0.584084	37	NFE2L2	60.40541
0.001883	0.546875	0.647147	37	HSPB1	61.83784
0.001037	0.522388	0.7	36	IL1A	54.19444
0.008251	0.545171	0.515873	36	ABCG2	53.63889
0.001953	0.540123	0.647059	35	IGFBP3	60.68571
0.001278	0.540123	0.709244	35	PARP1	57.8
0.005767	0.543478	0.384874	35	PPARA	46.65714
9.85E-04	0.538462	0.743316	34	RUNX2	65.67647
0.001474	0.538462	0.627451	34	RAF1	58.64706
0.003455	0.540123	0.460227	33	PRKCA	48.15152
0.00125	0.538462	0.668561	33	HSPA5	59.69697
0.002215	0.53681	0.534091	33	HSP90AB	51.21212
0.005318	0.543478	0.640152	33	GJA1	62.63636
0.00188	0.533537	0.711694	32	IGF2	62.34375
0.001183	0.517751	0.690323	31	TNFRSF1	54.83871
0.001401	0.527108	0.737634	31	SELP	57.80645
0.0024	0.531915	0.572043	31	NQO1	57.19355
0.001389	0.528701	0.703226	31	F3	57.51613
4.23E-04	0.530303	0.802299	30	CASP7	60
0.003583	0.519288	0.45977	30	PRKCD	47.16667
4.80E-04	0.514706	0.78836	28	CXCL2	58.21429
0.001758	0.525526	0.534392	28	SLC2A4	54.03571
0.007722	0.525526	0.320106	28	CYP3A4	36.32143
0.001801	0.525526	0.595442	27	NCF1	58.62963
0.002128	0.520833	0.475783	27	PRKCB	47.77778
0.002923	0.525526	0.433048	27	G6PD	47.59259
0.004067	0.519288	0.447293	27	CYP1A1	41.59259
6.65E-04	0.495751	0.744615	26	CHEK2	50
3.59E-04	0.513196	0.815385	26	IRF1	66.42308
0.018081	0.511696	0.452308	26	PRKACA	47.76923
0.002688	0.516224	0.476923	26	ABCA1	48.92308
8.40E-04	0.50578	0.58	25	CHUK	57.96
7.35E-04	0.507246	0.736667	25	E2F1	54.68
0.003116	0.523952	0.393333	25	RXRA	44.96

0.002391	0.517751	0.47	25	NCOA2	50.56
6.00E-04	0.488827	0.688406	24	TOP2A	51.70833
3.79E-04	0.50578	0.818841	24	PLAT	59.04167
3.19E-04	0.519288	0.841897	23	IL6R	65.17391
3.97E-04	0.49435	0.790514	23	BIRC5	56.08696
3.64E-04	0.528701	0.841897	23	ERBB3	72
0.001046	0.520833	0.72332	23	TOP1	60.86957
0.0047	0.517751	0.55336	23	DPP4	53.91304
0.009296	0.520833	0.482213	23	ENSG0000	56.6087
4.19E-04	0.483425	0.809524	22	THBD	53.95455
7.26E-04	0.508721	0.727273	22	IL2RA	58.31818
7.94E-04	0.507246	0.714286	22	ALOX5	62.81818
6.87E-04	0.513196	0.69697	22	CTSD	62.04545
0.001765	0.508721	0.489177	22	FASN	49.72727
4.15E-04	0.502874	0.895238	21	MMP10	70.14286
7.07E-04	0.495751	0.614286	21	BCL2	53.80952
4.67E-04	0.50578	0.752632	20	BAX	63.8
3.62E-04	0.467914	0.74269	19	CXCL11	50.15789
0.002383	0.498576	0.409357	19	PLA2G4A	47.78947
0.003124	0.507246	0.467836	19	AKR1B1	54.15789
9.60E-04	0.484765	0.372549	18	PRKCE	39.94444
0.001473	0.495751	0.496732	18	HK2	50.66667
0.00105	0.491573	0.535948	18	CYP1B1	41.16667
8.20E-04	0.49435	0.772059	17	COL1A2	61.35294
0.001228	0.486111	0.544118	17	GSTM1	39.17647
0.002611	0.469169	0.397059	17	CYP1A2	25.70588
0.00211	0.5	0.485294	17	NR1I2	43.76471
0.001256	0.483425	0.448529	17	PON1	41.76471
1.58E-04	0.460526	0.75	16	PCNA	43.3125
2.37E-04	0.492958	0.666667	16	RASA1	58.125
0.001406	0.483425	0.325	16	MGAM	38.6875
6.24E-04	0.495751	0.583333	16	UCP2	52.9375
3.92E-04	0.471698	0.685714	15	PTGS1	50.93333
5.47E-04	0.488827	0.561905	15	GSTM2	43.66667
3.92E-05	0.486111	0.87619	15	RASSF1	71.73333
5.12E-04	0.478142	0.609524	15	PTGES	52.13333
0.009762	0.504323	0.571429	15	ADRB2	61.4
0.001167	0.5	0.504762	15	ACHE	53.4
2.42E-04	0.459318	0.571429	14	PSMD3	53.07143
4.66E-04	0.446429	0.67033	14	COL3A1	47.21429
4.45E-05	0.465426	0.934066	14	E2F2	53.85714
5.23E-04	0.475543	0.626374	14	PTGER3	50.28571
4.56E-04	0.482094	0.571429	14	ACACA	41.78571
2.02E-04	0.487465	0.769231	13	ODC1	72.53846
4.05E-04	0.47043	0.615385	13	ABCG1	43.69231
4.88E-04	0.462963	0.30303	12	PPARD	40
7.70E-06	0.460526	0.984848	12	IL10RA	53.58333

6.43E-04	0.478142	0.69697	12	HSF1	56.33333
6.46E-05	0.487465	0.763636	11	NR3C2	64.36364
6.78E-04	0.472973	0.490909	11	ALDH3A1	39.27273
6.91E-04	0.436409	0.509091	11	NR1I3	30
1.70E-04	0.465426	0.666667	10	HAS2	54.4
2.15E-04	0.462963	0.666667	10	TNFAIP6	64.9
4.51E-04	0.448718	0.6	10	PTGES2	35
1.28E-04	0.455729	0.8	10	F10	43.6
8.05E-04	0.455729	0.733333	10	F7	45.3
1.37E-04	0.462963	0.638889	9	RXRG	47.11111
5.12E-05	0.474255	0.694444	9	PIK3CG	72.33333
0.001908	0.433168	0.25	8	KCNH2	26.625
1.25E-05	0.474255	0.857143	8	DUOX2	66.625
2.87E-05	0.458115	0.821429	8	NKX3-1	65.375
2.70E-04	0.436409	0.428571	7	GLB1	33.57143
1.11E-04	0.454545	0.666667	7	XDH	47.57143
0.002226	0.434243	0.142857	7	MAOB	24.85714
3.27E-04	0.398633	0.4	6	SCN5A	24.33333
2.40E-04	0.389755	0.4	6	ABCC4	20.83333
2.08E-05	0.435323	0.8	6	CETP	42.16667
2.49E-04	0.366876	0.5	5	KCNK2	13.4
2.99E-06	0.427873	0.9	5	ELK1	61
0	0.435323	1	5	PRSS1	62
2.66E-04	0.417661	0.1	5	ALDH2	29.4
4.63E-05	0.427873	0.5	4	INSRR	51.25
0.011429	0.431034	0.5	4	CLDN4	62.25
3.49E-06	0.443038	0.833333	4	RUNX1T1	77
5.51E-04	0.425791	0.333333	3	NPEPPS	45
1.26E-04	0.385463	0	2	EIF6	36
3.63E-05	0.343811	0	2	SLC6A2	11
0	0.339147	0	1	PKIA	26
0	0.301724	0	1	GABRA1	4
0	0.381264	0	1	ACPP	66
