# Supplementary material for: Network Pharmacology-Based Study on the Mechanism of Aloe Vera for Treating Cancer
Source: Evid Based Complement Alternat Med. 2021 Dec 1;2021:6077698. doi: 10.1155/2021/6077698 (PMC8654547; doi:10.1155/2021/6077698)
Supplement: Supplementary Materials — Supplementary Table S1: target analysis including degree values. Supplementary Table S2: complete output of molecular docking. [file 6077698.f1.zip › 6077698.f1/Table S2.pdf]

AKT1 & Quercetin

mode	affinity (kcal/mol)	dist from best mode rmsd l.b.	rmsd u.b.
1	-6.0	0.000	0.000
2	-5.9	16.585	18.530
3	-5.8	15.158	16.879
4	-5.7	13.953	17.155
5	-5.6	13.853	16.085
6	-5.6	1.978	3.864
7	-5.5	1.954	6.998
8	-5.5	14.854	17.752
9	-5.3	3.674	5.137
10	-5.3	4.694	8.688

AKT1 & Arachidonic acid

mode	affinity (kcal/mol)	dist from best mode rmsd l.b.	rmsd u.b.
1	-4.8	0.000	0.000
2	-4.7	4.242	7.757
3	-4.7	12.300	15.579
4	-4.6	4.120	7.796
5	-4.6	3.149	4.826
6	-4.6	2.978	4.892
7	-4.6	2.602	5.700
8	-4.5	4.360	7.456
9	-4.4	12.030	15.413
10	-4.4	4.998	8.516

AKT1 & Aloe-emodin

mode	affinity (kcal/mol)	dist from best mode rmsd l.b.	rmsd u.b.
1	-6.1	0.000	0.000
2	-6.1	1.126	2.951
3	-5.9	15.289	15.549
4	-5.8	15.252	16.807
5	-5.7	14.406	15.235
6	-5.5	14.750	16.390
7	-5.5	15.612	15.944
8	-5.5	1.651	3.888
9	-5.5	14.037	14.550
10	-5.4	1.970	5.128

AKT1 & Beta-Carotene

mode | affinity | dist from best mode

	(kcal/mol)	rmsd l.b.	rmsd u.b.
1	-7.0	0.000	0.000
2	-6.8	3.448	4.920
3	-6.7	2.102	3.255
4	-6.4	1.011	16.609
5	-6.3	2.315	3.574
6	-6.2	1.981	2.720
7	-6.2	0.836	16.455
8	-5.9	2.190	3.235
9	-5.8	2.614	16.693
10	-5.7	2.482	3.483

TP53 & Quercetin

mode	affinity	dist from best mode	
	(kcal/mol)	rmsd l.b.	rmsd u.b.
1	-8.2	0.000	0.000
2	-8.0	1.344	6.266
3	-7.9	3.484	5.476
4	-7.6	2.501	4.633
5	-7.6	1.985	6.787
6	-7.2	4.472	6.547
7	-7.2	5.186	7.787
8	-7.2	4.110	7.013
9	-7.1	3.114	7.039
10	-7.0	3.302	5.731

TP53 & Arachidonic acid

mode	affinity	dist from best mode	
	(kcal/mol)	rmsd l.b.	rmsd u.b.
1	-6.2	0.000	0.000
2	-6.1	1.631	2.618
3	-5.8	2.051	2.327
4	-5.7	2.263	5.087
5	-5.7	3.129	6.470
6	-5.7	6.607	9.088
7	-5.7	6.479	9.584
8	-5.7	5.880	10.161
9	-5.7	5.463	9.613
10	-5.7	5.021	7.463

TP53 & Aloe-emodin

mode	affinity	dist from best mode	
	(kcal/mol)	rmsd l.b.	rmsd u.b.

```
  1        -8.0      0.000      0.000
  2        -8.0      1.634      5.702
  3        -7.7      1.300      5.236
  4        -7.6      1.256      3.116
  5        -7.4      1.110      6.130
  6        -7.2      1.722      5.786
  7        -7.2      1.166      1.732
  8        -7.1      1.439      3.075
  9        -6.9      1.406      3.215
 10        -6.7      1.772      6.459
```

TP53 & Beta-carotene

```
mode |   affinity | dist from best mode
     | (kcal/mol) | rmsd l.b.| rmsd u.b.
-----+------------+----------+----------
  1        -7.4      0.000      0.000
  2        -7.0      0.805     16.706
  3        -6.7      1.456     16.929
  4        -6.5      1.346      2.138
  5        -6.0      1.131      1.792
  6        -5.8      1.051     16.974
  7        -5.1      1.435      2.400
  8        -4.4      6.116      8.448
  9        -4.4      1.965      3.304
```

VEGFA & Quercetin

```
mode |   affinity | dist from best mode
     | (kcal/mol) | rmsd l.b.| rmsd u.b.
-----+------------+----------+----------
  1        -5.6      0.000      0.000
  2        -5.6      3.064      4.787
  3        -5.3      1.311      2.919
  4        -5.2      2.579      4.067
  5        -5.1      2.792      5.617
  6        -5.1      1.790      6.455
  7        -4.9      9.030     12.324
  8        -4.9      2.644      6.631
  9        -4.8     10.468     11.292
 10        -4.8      9.591     11.944
```

VEGFA & Arachidonic acid

```
mode |   affinity | dist from best mode
     | (kcal/mol) | rmsd l.b.| rmsd u.b.
-----+------------+----------+----------
  1        -3.9      0.000      0.000
  2        -3.5      2.342      4.550
  3        -3.4      3.092      6.041
```

4	-3.4	2.159	3.461
5	-3.4	1.422	2.418
6	-3.4	8.177	11.436
7	-3.2	8.258	11.102
8	-3.2	8.404	11.506
9	-3.2	8.961	12.080
10	-3.1	2.701	5.464

VEGFA & Aloe_emodin

mode	affinity (kcal/mol)	dist from best mode rmsd l.b.	rmsd u.b.
1	-5.9	0.000	0.000
2	-5.4	2.817	4.373
3	-5.3	1.260	6.001
4	-5.2	1.261	3.045
5	-5.1	3.046	6.542
6	-5.0	2.804	3.598
7	-4.9	2.834	3.666
8	-4.8	2.958	6.485
9	-4.8	1.928	2.639
10	-4.8	2.842	6.134

VEGFA & Beta_Carotene

mode	affinity (kcal/mol)	dist from best mode rmsd l.b.	rmsd u.b.
1	-1.1	0.000	0.000
